# Supplementary material for: A diencephalic circuit in rats for opioid analgesia but not positive reinforcement
Source: Nat Commun. 2022 Feb 9;13:764. doi: 10.1038/s41467-022-28332-6 (PMC8828762; doi:10.1038/s41467-022-28332-6)
Supplement: Supplementary file 1 — Supplementary Information [file 41467_2022_28332_MOESM1_ESM.pdf]

Supplementary Figure 1

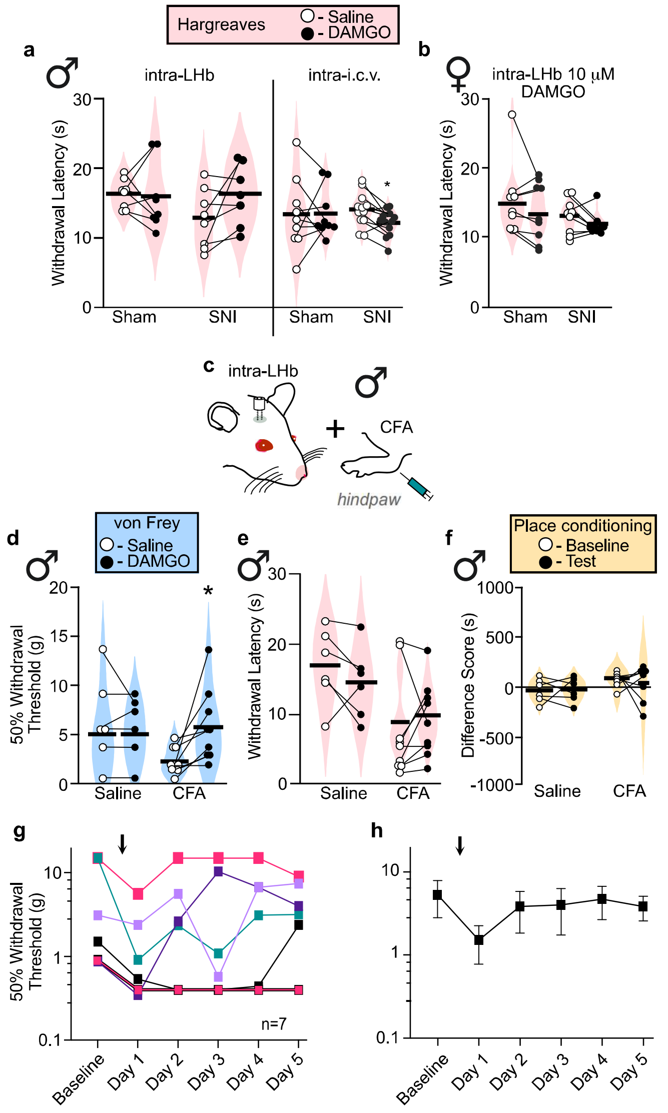

Supplementary Figure 1. *Further characterization of neuropathic pain and inflammatory pain models and the impact of intra-LHb MOR activation in rat.* **a,** (Left) Violin plots of withdrawal latency to heat in male sham (n = 9) and SNI (n = 8) rats with SNI after saline vs DAMGO (10 μM) microinjections into the LHb. Two-way mixed ANOVA revealed no significant interaction between injury and drug condition ($F(1,14) = 2.522$, $p = 0.135$) in the intra-LHb male cohort (Left). Bonferroni corrected paired t test was conducted between drug conditions for normally distributed Sham (Sham males: $p = .861$) and SNI (SNI males: $p = 0.096$) animals. Two-way mixed ANOVA indicated no significant interaction between injury and drug condition ($F(1,19) = 1.549$, $p = 0.228$) in male intra-i.c.v. cohort (Right). Sham animals were non-parametrically distributed, the Wilcoxon signed rank test was conducted ($V = 24$, $p = 0.9102$) while SNI animals were normally distributed, and a Bonferroni corrected paired t test was conducted ($p = 0.0433$). **b,** Two-way mixed ANOVA indicated no significant interaction between injury and drug condition ($F(1,16) = 0.008$, $p = 0.929$). Both the Sham and SNI female groups were non-parametrically distributed, the Wilcoxon signed rank test was conducted, both conditions yielding non-significant differences ($V = 13$, $p = 0.3008$). **c,** To test if the effects of MOR activation in the LHb generalize to other forms of pain, we repeated the manipulations above with an inflammatory model of pain. Male rats received an intradermal injection of CFA or sterile saline into the plantar aspect of the hindpaw. Schematic diagram of inflammatory injury preparations and cannulation targeting the LHb. **d**, Mechanical withdrawal thresholds in sham and CFA-injured rats. In animals with CFA, we observed an increase in the average withdrawal threshold following intra-LHb DAMGO (10 μM) compared to saline, indicating a reduction in mechanical allodynia: Two-way mixed ANOVA revealed significant injury and drug condition interaction ($F(1,13) = 5.772$, $p = 0.032$) with Bonferroni corrected paired t-test, SNI $p = 0.0178$; saline $p = 0.796$. **e**, Withdrawal latency to heat. DAMGO in the LHb did not reverse the CFA-induced decrease in withdrawal latency to heat. Two-way mixed ANOVA revealed no significant injury and drug condition interaction ($F(1,13) = 2.589$, $p = 0.132$). Bonferroni corrected pair t tests were conducted; saline: $p = 0.455$; CFA: Wilcoxon signed rank test, $V = 33$, $p = 0.25$. **f**, Rats with CFA injury also did not develop a preference to intra-LHb DAMGO in the place conditioning paradigm: Two-way mixed ANOVA, no significant interaction between CFA/saline and baseline/test $F(1,11) = 0.104$, $p = 0.753$. Bonferroni corrected t tests were conducted between drug conditions for saline (saline: $p = 0.901$) or CFA (CFA: $p = 0.706$) treated animals. The discrepancy between place conditioning results of our neuropathic and inflammatory pain animals may be due to the natural history of the injury caused in the CFA model: whereas SNI animals underwent permanent nerve ligation, animals injected with CFA presented with transient swelling and erythema of the hindpaw. Within a few days of CFA injection, this phenotype had resolved in our rats. **g,** Mechanical allodynia also reversed quickly: in a separate cohort of rats, all subjects displayed a sharp decline in withdrawal threshold from baseline on day 1 following CFA injection, but the injury-induced allodynia returned to control levels by day 5 in many animals. Individual animals were tested daily; each line represents a male rat. **h,** Average across rats shows that our CFA-induced reduction in mechanical allodynia reversed within a few days. Since our place conditioning training exceeds this time window, resolution of the injury probably occurred before training was completed, thus preventing the formation of a robust association between pain relief and place conditioning apparatus context to produce a CPP. Circles or squares indicate individual animals, while connecting lines indicate within-subject manipulations. $^{*}p \leq 0.05$

Supplementary Figure 2

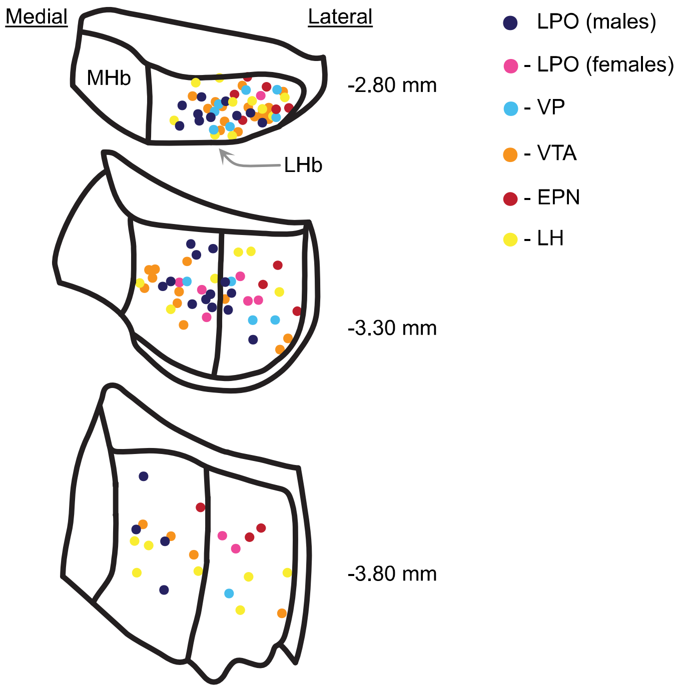

Supplementary Figure 2. *Neurons receiving functional synaptic inputs from the five brain regions showing connectivity were distributed throughout the LHb.* Locations of neurons with functional synaptic connections detected using whole-cell recordings from optogenetic stimulation of terminals arising from each input tested. Locations are based on a combination of biocytin immunohistochemistry localization and low magnification visualization of the cell location during recording. Color indicates source of innervation.

Supplementary Figure 3

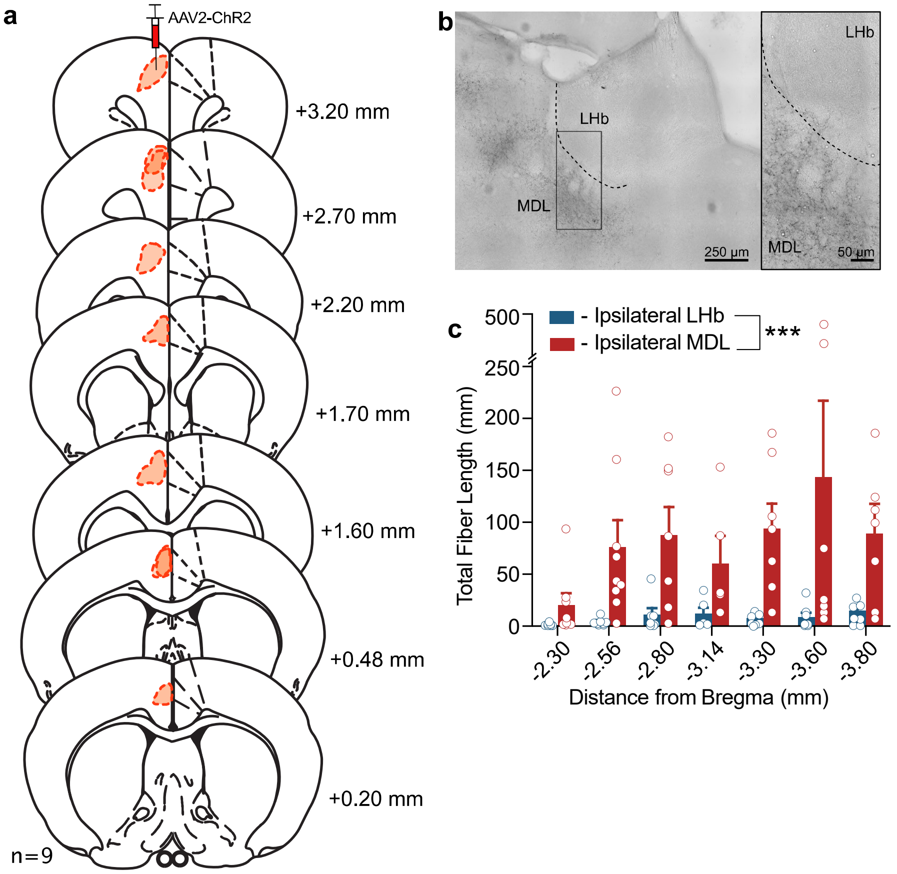

Supplementary Figure 3. *ACC minimally innervates the LHb.* **a**, Diagram of the extent of unilateral AAV2-hSyn-hChR2(H134R)-mCherry injection sites (n = 9 male rats) throughout anteroposterior range of the ACC for anterograde tracing study. **b,** Example ipsilateral DAB-positive fibers (black) visualized under brightfield illumination. Fibers heavily innervate the MDL, which abuts the lateral edge of the LHb, while sparse to no fibers innervate the LHb. Contralateral innervation of LHb and MDL was negligible compared to the ipsilateral side, and therefore were omitted from our analysis. (Left) Scale bar = 250 µm. (Right) Scale bar = 50 µm. **c**, Average stereologically-quantified DAB-positive fiber lengths throughout anteroposterior range of the LHb, compared to the MDL innervation in the same coronal slice. Data are indicated as bar graphs with mean ± SEM, and circles as individual animals. Mann-Whitney test, two-tailed: $U = 0$, $p = 0.0006$.

Supplementary Figure 4

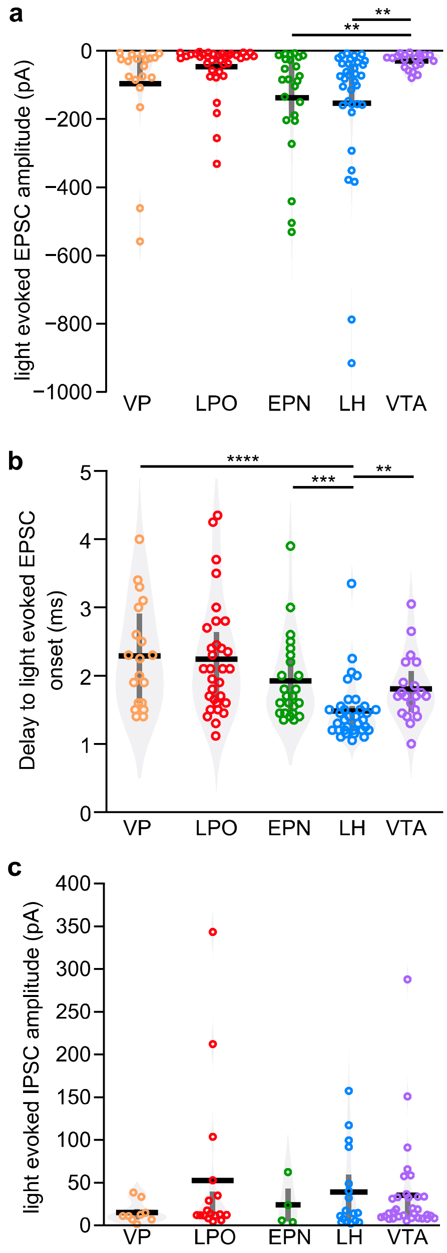

Supplementary Figure 4. *Sources of glutamatergic inputs to the rat LHb vary in strength and delay in synaptic transmission.* **a**, We observed some variations between inputs in the mean light evoked EPSCs (V_m = -60 mV). In particular, excitatory inputs from the VTA were consistently small: Kruskal-Wallis χ2 = 25.5, df = 4, p = 0.00004, followed by Dunn Test for pairwise comparisons. **b**, Differences were also detected in the delay to the onset of light evoked EPSCs to LHb neurons (circles) from these different sources, with inputs from LH showing the fastest response. Although these data deviate from a normal distribution (Shapiro's test, p = 0.0000001), KDEs (violins) are consistent with continuous distributions, suggesting reliable polysynaptic events were rarely detected. **c**, We observed some variations between inputs in the mean light evoked IPSCs (V_m = -40 mV). While there were no statistically significant differences in amplitudes detected, the mean inhibitory input from the VP was particularly small: Kruskal-Wallis χ2 = 2.3, df = 4, p = 0.7. ** p < 0.01, *** p < 0.005, **** p < 0.0005

Supplementary Figure 5

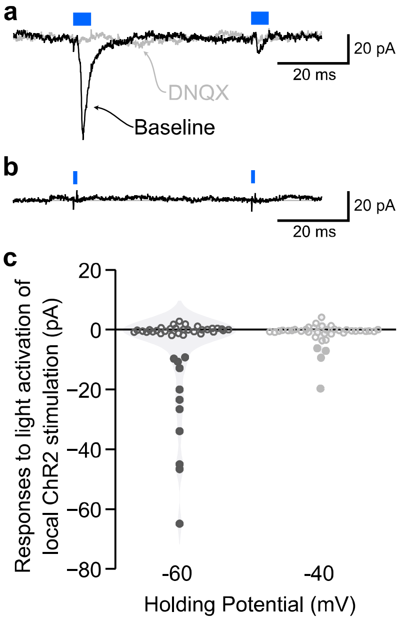

Supplementary Figure 5. *Optogenetic experiments detect local glutamate, but not GABA, functional connections in rat LHb.* AAV2-hSyn-hChR2(H134R)-mCherry was stereotaxically injected into the LHb at least 4 weeks prior to *ex vivo* whole cell recordings in the LHb to detect local synaptic connections. Neurons were recorded blind to ChR2 expression, therefore in some cases the patched neuron expressed ChR2. Therefore, in addition to connectivity criteria used for other afferent inputs, in these experiments only light evoked inward currents that were blocked by 10 µM DNQX were considered glutamatergic connections, and direct ChR2 induced inward currents were subtracted out for the quantification illustrated here. Each cell was probed for both glutamate and GABA inputs in voltage clamp by holding neurons at V_m = -60 mV and -40 mV, light pulse durations 1, 5, and 10 ms durations. **a**, Example recording at V_m = -60 mV showing a light evoked response that was blocked by DNQX. **b**, Example recording at V_m = -40 mV with minimal outward current response within 7 ms of light pulse. This example is the largest outward deflection from baseline observed in these experiments. **c**, Summary of all LHb recordings tested in this experiment. Filled circles represent cells where responses could be classified as local ChR2 induced synaptic transmission. When no clear response was detected, the measure indicated is the difference between the mean $I_{holding}$ of the baseline 100 ms period just prior to the light pulse and the mean $I_{holding}$ 2 ms period starting 2 ms after initiation of the light pulse, consistent with monosynaptic delay timing. None of the recordings made at V_m = -40 mV met the criteria for a response to light stimulation with an outward current.

Supplementary Figure 6

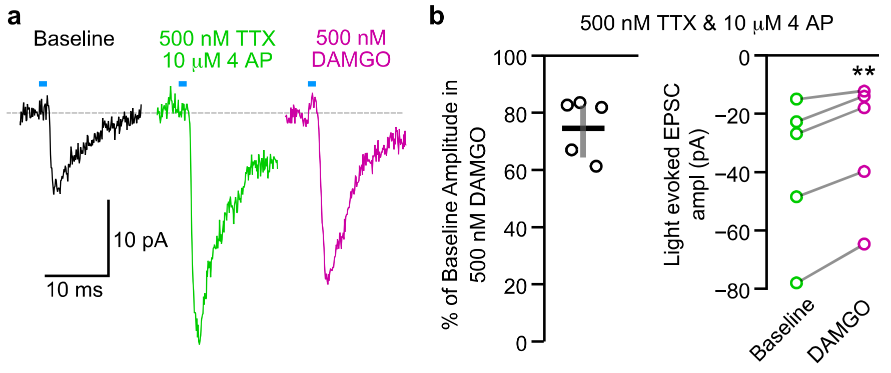

Supplementary Figure 6. *Isolated monosynaptic glutamatergic inputs from LPO to LHb neurons are inhibited by MOR activation.* **a,** Example light evoked EPSC responses in an LHb neuron from a rat with ChR2 expression in LPO neurons. This response persisted in monosynaptic isolation by 500 nM TTX and 100 µM 4 AP (green), and this isolated response was inhibited by 500 nM DAMGO (magenta). **b**, Summary of DAMGO effects on isolated monosynaptic EPSC inputs to LHb neurons (circles) expressed as % of baseline monosynaptic response (left) and as raw EPSC magnitudes (right). Paired t-test, df = 4, t = -5.1, p = 0.007. ** p < 0.01

Supplementary Figure 7

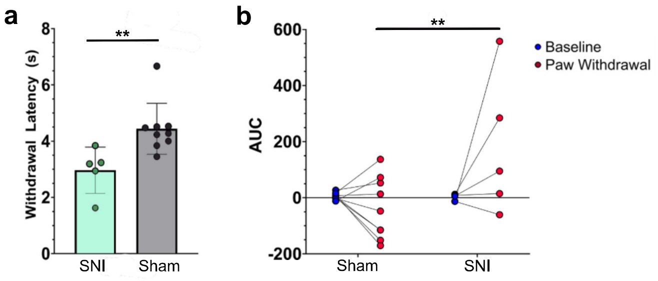

Supplementary Figure 7. *Mice with SNI show hypersensitivity to heat and increased activity in LHb-projecting LPO neurons during paw withdrawal from thermal stimulation.* **a,** Mice with SNI show a reduced latency to withdraw their paw following thermal stimulation in the Hargreaves task. Unpaired t-test, $t(12) = 3.007$, $p = 0.011$. **b,** VGluT2-expressing LPO neurons that project to the LHb expressed GCaMP6m and showed a greater calcium response during paw withdrawal to Hargreaves thermal stimulation (area under the curve, deviation from baseline fluorescence) in SNI animals ($n = 5$) compared to sham controls ($n = 9$): Two-way ANOVA, $F(1,12) = 5.4$, $p = 0.038$; Holm-Sidak post-hoc test, $p = 0.0074$. Data are indicated as bar graphs with mean ± SEM, and circles as individual animals. ** $p < 0.01$

Supplementary Figure 8

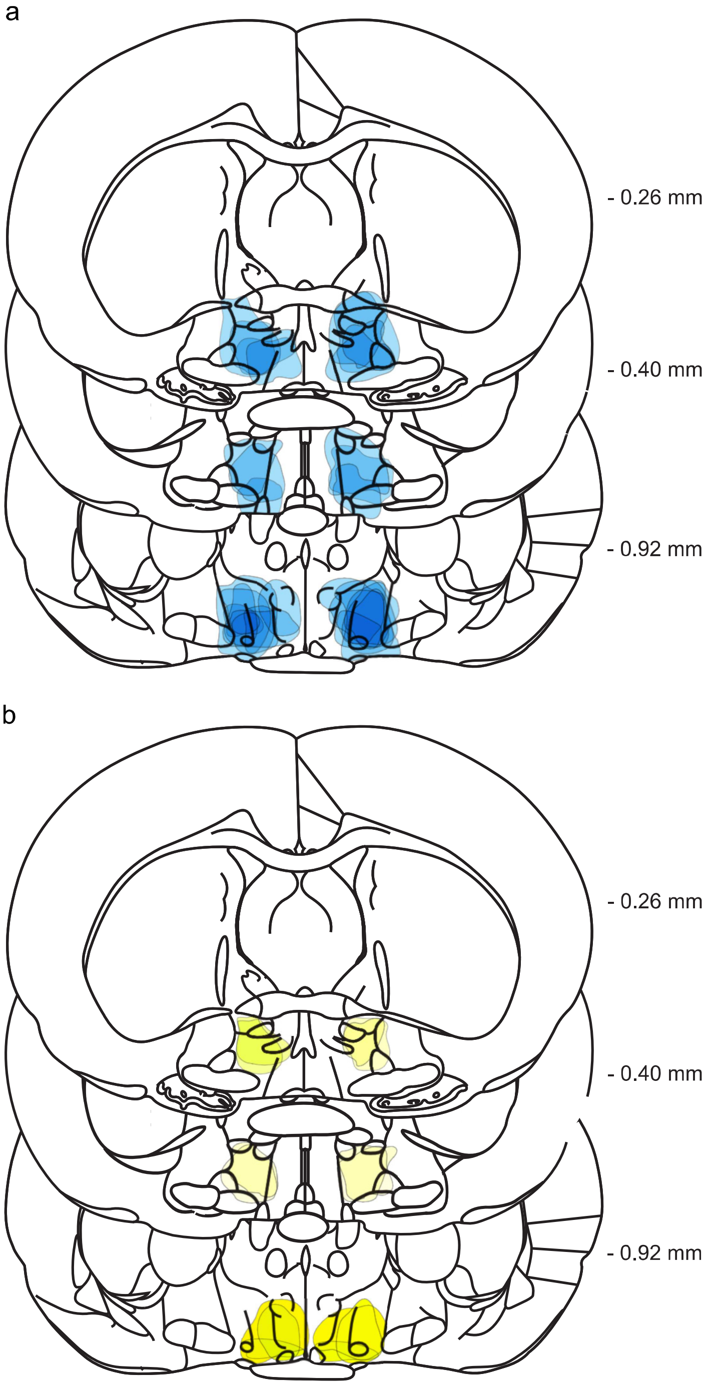

Supplementary Figure 8. Locations and spread of bilateral ChR2 (a) and iC++ (b) expression in the LPO in rats used for *in vivo* optogenetic experiments in Fig 5 and 6.

Supplementary Table 1: Assumption testing on behavioral data

Experiment	Figure	# animals; # Outliers; # extreme outliers	Shapiro-Wilk Test of Normality (across all groups) Statistic; p val	Test for Homogeneity of Variances<br><br>Statistic; p val (Test used)	Parametric test	Non-parametric test: Repeated measures Wilcoxon signed rank exact test<br><br>V; p val
von Frey: Male; Sham; 10 μM DAMGO in LHb	1b	8; 0; 0	Saline: 0.807; 0.0340<br>DAMGO: 0.743; 0.00687	1.46; 0.248 (Levene's test)	n/a	4; 0.8551
von Frey: Male; SNI; 10 μM DAMGO in LHb	1b	8; 0; 0	Saline: 0.814; 0.0407<br>DAMGO: 0.853; 0.102	1.49; 0.242 (Levene's test)	n/a	26; 0.05024
von Frey: Male; Sham; 10 μM DAMGO in i.c.v.	1b	9; 0; 0	Saline: 0.812; 0.0283<br>DAMGO: 0.701; 0.00144	0.0897; 0.768 (Levene's test)	n/a	7; 0.5294
von Frey: Male; SNI; 10 μM DAMGO in i.c.v.	1b	12; 2; 2	Saline: 0.660; 0.000358<br>DAMGO: 0.790; 0.00726	0.168; 0.686 (Levene's test)	n/a	31; 0.08006
von Frey: Female; Sham; 10 μM DAMGO in LHb	1c	9; 0; 0	Saline: 0.857; 0.0890<br>DAMGO: 0.875; 0.137	0.0709; 0.793 (Levene's test)	Paired t-test, Bonferroni adjusted p = 0.703	n/a
von Frey: Female; SNI; 10 μM DAMGO in LHb	1c	9; 2; 1	Saline: 0.565; 0.0000375<br>DAMGO: 0.845; 0.0655	1.82; 0.196 (Levene's Test)	n/a	7; 0.07422
von Frey: Female; Sham; 100 μM DAMGO in LHb	1c	6; 0; 0	Saline: 0.801; 0.0604<br>DAMGO: 0.912; 0.452	0.493; 0.499 (Levene's test)	Paired t-test, Bonferroni adjusted p = 0.92	n/a
von Frey: Female; SNI; 100 μM DAMGO in LHb	1c	8; 1; 1	Saline: 0.692; 0.00181<br>DAMGO: 0.811; 0.0379	1.13; 0.306 (Levene's test)	n/a	6; 0.4017
Place Conditioning: Male Sham/SNI; 10 μM DAMGO in LHb	1d	14; 1; 0	Sham x Baseline: 0.923; 0.453<br>SNI x Baseline: 0.943 ; 0.686<br>Sham x Test: 0.973; 0.921<br>SNI x Test: 0.840; 0.129	Bartlett's K-squared = 8.0891, df = 3, p-value = 0.04421 (Bartlett test)	Two-way mixed design ANOVA two-way interaction F(1,12) = 8.921 p = 0.011<br><br>effect of group on baseline: F = 0.418; adjusted p = .53<br>effect of group on test day: F = 4.35; adjusted p = 0.059<br><br>Paired t-tests, Bonferroni adjusted<br>Sham: adjusted p = 0.743<br>SNI: adjusted p = 0.014	n/a
Place Conditioning: Sham vs. Male SNI; 10 μM DAMGO in i.c.v.	1d	21; 2; 0	Sham x Baseline: 0.901; 0.257<br>SNI x Baseline: 0.967; 0.872<br>Sham x Test: 0.971; 0.905<br>SNI x Test: 0.985; 0.997	23.9; 0.000000997 (Box M-test)	Two-way mixed design ANOVA two-way interaction F(1,19) =2.239; p = 0.151<br><br>effect of group on baseline: F = 3.02; adjusted p = 0.099<br>effect of group on test day: F = 4.13; adjusted p = 0.056<br><br>Paired t-tests for non-significant two-way interaction Bonferroni adjusted:<br>Sham: adjusted p = 0.259<br>SNI: adjusted p = 0.489	n/a

Place Conditioning: Female Sham/SNI; 10 µM DAMGO in LHb	1e	18; 2; 0	Sham x Baseline: 0.959; 0.791<br>SNI x Baseline: 0.987; 0.990<br>Sham x Test: 0.871; 0.127<br>SNI x Test: 0.947; 0.665	Bartlett's K-squared = 18.085, df = 3, p-value = 0.0004224 (Bartlett test)	Two-way mixed design ANOVA two-way interaction F(1,16) =0.703; p = 0.414<br><br>effect of group on baseline: F = 0.002; adjusted p = 1<br><br>effect of group on test day: F = 0.481;<br>adjusted p = 0.996<br><br>Paired t-tests for non-significant two-way interaction Bonferroni adjusted:<br>Sham: adjusted p = 0.926<br>SNI: adjusted p = 0.0948	n/a
Place Conditioning: Female Sham/SNI; 100 µM DAMGO in LHb	1e	15; 3; 0	Sham x Baseline: 0.904 0.66<br>SNI x Baseline: 0.919; 0.384<br>Sham x Test: 0.87; 0.226<br>SNI x Test: 0.900; 0.250	8.59; 0.00339 (Box M-test)	Two-way mixed design ANOVA two-way interaction F(1,13) =6.234; p = 0.027<br><br>effect of group on baseline: F = 0.006; adjusted p = 1<br>effect of group on test day: F = 5.56; adjusted p = 0.07<br><br>Paired t-tests for non-significant two-way interaction Bonferroni adjusted:<br>Sham: adjusted p = 0.476<br>SNI: adjusted p = 0.0349	n/a
Place Conditioning; Male; ChR2 in LPO to LHb projections	5c	8; 0; 0	0.925; 0.208	4.31, .056 (Levene's test)	Paired t-test<br>Df = 7, t = -4.64, p = 0.002	n/a
Place Conditioning; Male; mCherry vs. ChR2; 10 µM DAMGO in LHb	5d	16; 0; 0	mCherry x Baseline: 0.941; 0.618<br>ChR2 x Baseline: 0.913; 0.374<br>mCherry x Test: 0.914; 0.385<br>ChR2 x Test: 0.847; 0.0891	18.3; 0.0000186 (Box M-test)	Two-way mixed design ANOVA two-way interaction, F(1,14) = 9.982; p = 0.007<br><br>effect of group on baseline: F = 0.01; adjusted p = 1<br>effect of group on test day: F = 12.5; adjusted p = 0.006<br><br>Paired t-tests for non-significant two-way interaction Bonferroni adjusted:<br>mCherry: adjusted p = 0.427<br>ChR2: adjusted p = 0.0027	n/a
Place Conditioning; Male; iC++ in LPO to LHb projections	6c	12; 0; 0	Sham x baseline: 0.868; 0.259<br>SNI x baseline: 0.985; 0.981<br>Sham x test: 0.860; 0.23<br>SNI x test: 0.938; 0.623	Bartlett's K-squared = 5.6657, df = 3, p-value = 0.1291 (Bartlett test)	Two-way mixed design ANOVA two-way interaction F(1,10) =3.443; p = 0.093<br><br>effect of group on baseline: F = 0.006; adjusted p = 1<br>effect of group on test day: F =4.26; adjusted p = .132<br><br>Paired t-tests for non-significant two-way interaction Bonferroni adjusted:<br>Sham: adjusted p = 0.535<br>SNI: adjusted p = 0.048	n/a
Fiber photometry; Sham vs. SNI mice	6e	14; not determined	not determined	not determined	Two-way mixed design ANOVA F(1,12) = 5.439;<br>p= 0.038;<br><br>Holm-Sidak post-hoc test, p = 0.0074	n/a
Hargreaves: Male; Sham; 10 µM DAMGO in LHb	SI 1a	8; 0 ; 0	Saline: 0.913; 0.376<br>DAMGO: 0.833 0.0633	Bartlett's K-squared = 2.789, df = 1, p-value = 0.09487 (Bartlett Test)	Paired t-test, Bonferroni adjusted p = 0.861	n/a

Hargreaves; Male; SNI; 10 µM DAMGO in LHb	SI 1a	8; 0; 0	Saline: 0.947; 0.680<br>DAMGO: 0.941; 0.622	Bartlett's K-squared = 3.704 df = 1, p-value = 0.096 (Bartlett Test)	Paired t-test, Bonferroni adjusted p = 0.132	n/a
Hargreaves: Male; Sham; 10 µM DAMGO in i.c.v.	SI 1a	9; 3; 0	Saline: 0.959; 0.789<br>DAMGO: 0.810 0.0262	0.840; 0.373 (Levene's test)	n/a	24; 0.9102
Hargreaves; Male; SNI; 10 µM DAMGO in i.c.v.	SI 1a	12; 0; 0	Saline: 0.951; 0.655<br>DAMGO: 0.937; 0.456	0.605; 0.445 (Levene's test)	Paired t-test, Bonferroni adjusted p = 0.0433	n/a
Hargreaves; Female; Sham; 10 µM DAMGO in LHb	SI 1b	9; 1;0	Saline: 0.755; 0.00622<br>DAMGO: 0.894; 0.217	0.0458; 0.833 (Levene's test)	n/a	13; 0.3008
Hargreaves; Female; SNI; 10 µM DAMGO in LHb	SI 1b	9; 1; 1	Saline: 0.928; 0.466<br>DAMGO: 0.649; 0.000358	2.47 ; 0.136 (Levene's test)	n/a	13; 0.3008
von Frey; Male; Saline; 10 µM DAMGO in LHb	SI 1d	6; 0; 0	Saline: 0.960; 0.818<br>DAMGO: 0.941; 0.666	0.166 ; 0.692 (Levene's test)	Paired t-test, Bonferroni adjusted p = 0.796	n/a
von Frey; Male; CFA; 10 µM DAMGO in LHb	SI 1d	9; 0; 0	Saline: 0.904 0.275<br>DAMGO: 0.893; 0.217	3.80; 0.0691 (Levene's test)	Paired t-test, Bonferroni adjusted p = 0.0178	n/a
Hargreaves; Male; Saline; 10 µM DAMGO in LHb	SI 1e	6; 0; 0	Saline: 0.961; 0.824<br>DAMGO: 0.964; 0.853	0.07; 0.796 (Levene''s test)	Paired t-test, Bonferroni adjusted p = 0.455	n/a
Hargreaves; Male; CFA; 10 µM DAMGO in LHb	SI 1e	9; 2; 2	Saline: 0.731; 0.00327<br>DAMGO: 0.956; 0.753	0.0461 ;0.833 (Levene's test)	n/a	33; 0.25
Place Conditioning; Male; Saline vs. CFA; 10 µM DAMGO in LHb	SI 1f	13; 1; 0	Saline x Baseline: 0.957; 0.795<br>CFA x Baseline: 0.940; 0.635<br>Saline x Test: 0.933; 0.605<br>CFA x Test: 0.828; 0.077	Bartlett's K-squared = 11.713, df = 3, p-value = 0.008434	Two-way mixed design ANOVA two-way interaction F(1,11) =0.104; p = 0.753<br><br>effect of group on baseline: F = 4.18; adjusted p = .132<br>effect of group on test day: F = 0.258; adjusted p = 1<br><br>Paired t-tests for non-significant two-way interaction Bonferroni adjusted:<br><br>Saline: adjusted p = 0.901<br>CFA: adjusted p = 0.706	n/a
ACC fiber innervation; Ipsi LHb vs. Ipsi MDL	SI 3c	9; 0; 0	not determined	not determined	n/a	Mann-Whitney test, two-tailed, U=0; p = 0.0006
Hargreaves; Sham vs. SNI mice	SI 7a	14; 3; 2	0.896; 0.0994	0.0012; 0.973 (Levene's test)	Unpaired t-test<br>Df = 12, t = 3.007, p = 0.0109	n/a
AUC summary: Hargreaves/Fiber photometry; Sham vs. SNI mice	SI 7b	14; 0; 0	Sham x Baseline: 0.979; 0.257<br>Sham x paw withdrawal: 0.932; 0.496<br>SNI x Baseline: 0.813; 0.103<br>SNI x paw withdrawal: 0.921; 0.538	53.4; 2.71 e-13 (Box M-test)	Two-way mixed design ANOVA F(1,12) = 5.439; p = 0.038; Holm-Sidak post-hoc test, p=0.0074	n/a
