# Supplementary material for: A diencephalic circuit in rats for opioid analgesia but not positive reinforcement
Source: Nat Commun. 2022 Feb 9;13:764. doi: 10.1038/s41467-022-28332-6 (PMC8828762; doi:10.1038/s41467-022-28332-6)
Supplement: Supplementary file 3 — Reporting Summary [file 41467_2022_28332_MOESM3_ESM.pdf]

nature portfolio

Corresponding author(s): Elyssa B Margolis

Last updated by author(s): Dec 16, 2021

Reporting Summary

Nature Portfolio wishes to improve the reproducibility of the work that we publish. This form provides structure for consistency and transparency in reporting. For further information on Nature Portfolio policies, see our Editorial Policies and the Editorial Policy Checklist.

Statistics

For all statistical analyses, confirm that the following items are present in the figure legend, table legend, main text, or Methods section.

n/a	Confirmed	
☐	☒	The exact sample size (*n*) for each experimental group/condition, given as a discrete number and unit of measurement
☐	☒	A statement on whether measurements were taken from distinct samples or whether the same sample was measured repeatedly
☐	☒	The statistical test(s) used AND whether they are one- or two-sided<br>*Only common tests should be described solely by name; describe more complex techniques in the Methods section.*
☐	☒	A description of all covariates tested
☐	☒	A description of any assumptions or corrections, such as tests of normality and adjustment for multiple comparisons
☐	☒	A full description of the statistical parameters including central tendency (e.g. means) or other basic estimates (e.g. regression coefficient) AND variation (e.g. standard deviation) or associated estimates of uncertainty (e.g. confidence intervals)
☐	☒	For null hypothesis testing, the test statistic (e.g. *F*, *t*, *r*) with confidence intervals, effect sizes, degrees of freedom and *P* value noted<br>*Give P values as exact values whenever suitable.*
☒	☐	For Bayesian analysis, information on the choice of priors and Markov chain Monte Carlo settings
☒	☐	For hierarchical and complex designs, identification of the appropriate level for tests and full reporting of outcomes
☐	☒	Estimates of effect sizes (e.g. Cohen's *d*, Pearson's *r*), indicating how they were calculated

Our web collection on statistics for biologists contains articles on many of the points above.

Software and code

Policy information about availability of computer code

Data collection	For place conditioning experiments, either MED PC IV or custom software was utilized to track position indicated by beam breaks per second. Video tracking of animals in the custom made place preference apparatus was accomplished using Logitech webcam and Viewer Biobserve software.
Data analysis	For fiber photometry data analysis was conducted using custom written MATLAB scripts reported in Bruno et al. (2021) and available at https://github.com/djamesbarker/FiberPhotometry. For behavioral data analysis, GraphPad Prism 9 or R Studio 1.4 was used. Electrophysiology data was analyzed in Python 3.10. Stereology was conducted using MBF Stereoinvestigator 2020.Data was collated in excel for microsoft 365 for source data.

For manuscripts utilizing custom algorithms or software that are central to the research but not yet described in published literature, software must be made available to editors and reviewers. We strongly encourage code deposition in a community repository (e.g. GitHub). See the Nature Portfolio guidelines for submitting code & software for further information.

Data

Policy information about availability of data

All manuscripts must include a data availability statement. This statement should provide the following information, where applicable:

- Accession codes, unique identifiers, or web links for publicly available datasets
- A description of any restrictions on data availability
- For clinical datasets or third party data, please ensure that the statement adheres to our policy

The data generated in this study have been deposited in the Open Science Framework database [https://osf.io/mwyb3/?

view_only=28fcc13c3cac41a7a55e27ffccd62bda]. The raw data are protected and are not available due to data privacy laws. The processed data are available at the Open Science Framework database. The data generated in this study are also provided in the Supplementary Information/Source Data file.

Field-specific reporting

Please select the one below that is the best fit for your research. If you are not sure, read the appropriate sections before making your selection.

☒ Life sciences ☐ Behavioural & social sciences ☐ Ecological, evolutionary & environmental sciences

For a reference copy of the document with all sections, see nature.com/documents/nr-reporting-summary-flat.pdf

Life sciences study design

All studies must disclose on these points even when the disclosure is negative.

Sample size	Power analysis was conducted (Power = 0.80, alpha = 0.05, effect size = 15-30%) based on pilot studies and previous place preference studies using similar cannulation techniques (Waung et al. 2019, Mitchell et al. 2013). For immunohistochemical studies, at least three animals were used for cell counting. For ACC-LHb innervation, at least three animals for anterior, middle and posterior ACC were used to develop a comprehensive map. For electrophysiological studies, no power analysis was conducted, but a minimum of 2 animals were used to generate slices and the number of cells required were determined based off of previous electrophysiological studies from the lab (Waung et al. 2019).
Data exclusions	Pre-established exclusion criteria was used to remove animals from behavior studies. Specifically, if any animal displayed a significant baseline place preference defined as spending greater than 65% of total time on one chamber, they were excluded from the study. Animals that underwent surgery were excluded for three major reasons: 1) failure to develop hypersensitivity after spared nerve injury (>90% baseline sensory testing response), 2) off target injection or cannula placement and 3) animals with surgical complications as determined by animal care guidelines and veterinary consultation at the University of California, San Francisco.
Replication	Generally, rat behavioral cohorts were conducted in parallel (sham vs. injury) across 3 cohorts of animals except for the case of the i.c.v injection where 2 cohorts of animals were run. For mice studies, no replication was conducted as all animals were tested in parallel.For immunohistochemistry, all tissue was tested in parallel, but not replicated due to the comprehensive assessment across the entire brain region for each animal. For electrophysiology studies, multiple neurons were tested, from slices generated from at least two different rats. Main effects were consistent across replications, suggesting successful replication.
Randomization	Rats were initially grouped house 3 to a cage. Animals were randomly sampled across different cages for each cohort, For place preference studies, laser stimulation or drug infusion was pseudorandomly assigned and counterbalanced by chamber side and time of day. For sensory testing, drug infusion order (saline or DAMGO) was counterbalanced. For innervation studies, stereological analysis was conducted pseudorandomly by computer dictated grid presentation. For electrophysiological studies, animals previously underwent surgery, where they were sampled across multiple cages.
Blinding	All behavior was conducted blind to animal condition (pain vs. sham), virus, or infusion composition. Electrophysiological experiments were conducted blind to injection site. For immunohistochemical analysis, experiments were conducted blind to injection site.

Reporting for specific materials, systems and methods

We require information from authors about some types of materials, experimental systems and methods used in many studies. Here, indicate whether each material, system or method listed is relevant to your study. If you are not sure if a list item applies to your research, read the appropriate section before selecting a response.

Materials & experimental systems

n/a	Involved in the study	
☐	☒	Antibodies
☒	☐	Eukaryotic cell lines
☒	☐	Palaeontology and archaeology
☐	☒	Animals and other organisms
☒	☐	Human research participants
☒	☐	Clinical data
☒	☐	Dual use research of concern

Methods

n/a	Involved in the study	
☒	☐	ChIP-seq
☒	☐	Flow cytometry
☒	☐	MRI-based neuroimaging

Antibodies

Antibodies used	For the ACC innervation study, Rabbit anti-mCherry (1:5000, Ab167453, Abcam) and biotinylated goat anti-rabbit (1:200, BA1000, Vector Laboratories) antibodies were used. For retrograde tracing, rabbit anti FG (1:500, AB153, Millipore) and biotinylated goat anti-rabbit ((1:200, BA1000, Vector Laboratories) antibodies were used.
Validation	1. Rabbit anti-mCherry antibody: validated per manufacturer's website where antibody staining is only seen in HEK293 cells expressing mCherry. Cited by Howe et al. 2011 and Anacker et al. 2018

	2. Rabbit anti-FG antibody: validated per manufacturers webpage. Successful detection of FG was found in 1:10,000 dilution in cerebral cortex tissue. Cited by Barker et al. 2017

Animals and other organisms

Policy information about studies involving animals; ARRIVE guidelines recommended for reporting animal research

Laboratory animals	Male and Female Sprague Dawley rats were obtained from Charles River, surgeries were conducted at4-5 weeks of age and behavior/electrophysiology was conducted at 8-12 weeks of age. VGLuT2:cre mice were obtained from Jackson Laboratory (016963), mice 6-12 weeks of age were used. Rats were allowed access to food and water ad libitum and maintained on a 12h:12h light/dark cycle. Rats used in behavioral and in situ hybridization studies were housed under reverse light/dark cycle conditions. Rats were group housed until they underwent surgery, after which they were singly housed. Mice were allowed access to food and water ad libitum and maintained on a 12h:12h light/dark cycle with lights on at 7 AM. Mice were always housed in groups of 2-5. All animals were kept at an ambient temperature ranging from 65-75 degrees F, with humidity ranging from 50-60%.
Wild animals	Study did not involve wild animals
Field-collected samples	study did not involve field-collected samples
Ethics oversight	All experiments were performed in accordance with the guidelines of the National Institutes of Health Guide for the Care and Use of Laboratory Animals and the Institutional Animal Care and Use Committees (IACUC) at the University of California San Francisco, the National Institute on Drug Abuse (NIDA), and Rutgers University.

Note that full information on the approval of the study protocol must also be provided in the manuscript.
